# Magnetic Excitations in Ferromagnetically Coupled Spin‐1 Nanographenes

**DOI:** 10.1002/anie.202412353

**Published:** 2024-11-06

**Authors:** Elia Turco, Fupeng Wu, Gonçalo Catarina, Nils Krane, Ji Ma, Roman Fasel, Xinliang Feng, Pascal Ruffieux

**Affiliations:** ^1^ Empa – Swiss Federal Laboratories for Materials Science and Technology nanotech@surfaces Laboratory 8600 Dübendorf Switzerland; ^2^ Max Planck Institute of Microstructure Physics Weinberg 2 06120 Halle Germany; ^3^ Center for Advancing Electronics Dresden (cfaed) & Faculty of Chemistry and Food Chemistry Technische Universität Dresden Mommsenstrasse 4 01062 Dresden Germany; ^4^ Department of Chemistry Biochemistry and Pharmaceutical Sciences University of Bern 3012 Bern Switzerland

**Keywords:** ferromagnetism, nanographenes, Heisenberg model, triangulenes, scanning probe microscopy

## Abstract

In the pursuit of high‐spin building blocks for the formation of covalently bonded 1D or 2D materials with controlled magnetic interactions, π ‐electron magnetism offers an ideal framework to engineer ferromagnetic interactions between nanographenes. As a first step in this direction, we explore the spin properties of ferromagnetically coupled triangulenes—triangular nanographenes with spin S=1 . By combining in‐solution synthesis of rationally designed molecular precursors with on‐surface synthesis, we successfully achieve covalently bonded S=2 triangulene dimers and S=3 trimers on Au(111). Starting with the triangulene dimer, we meticulously characterize its low‐energy magnetic excitations using inelastic electron tunneling spectroscopy (IETS). IETS reveals conductance steps corresponding to a quintet‐to‐triplet excitation, and a zero‐bias peak resulting from higher‐order spin‐spin scattering of the five‐fold degenerate ferromagnetic ground state. The Heisenberg model captures the key parameters of inter‐triangulene ferromagnetic exchange, and its successful extension to the larger S=3 system validates the model's accuracy. We anticipate that incorporating ferromagnetically coupled building blocks into the repertoire of magnetic nanographenes will unlock new possibilities for designing carbon nanomaterials with complex magnetic ground states.

## Graphical abstract

On‐surface synthesis of high‐spin *S*=2 and *S*=3 nanographenes achieved through covalent linking of triangulenes via 1,3‐ and 1,3,5‐phenylene spacers is reported. Using scanning tunneling spectroscopy, magnetic evidence of their degenerate ground states and an effective ferromagnetic inter‐triangulene exchange is established. A spin model using a single coupling parameter describes magnetic excitations in both, the *S*=2 and *S*=3 nanographenes.

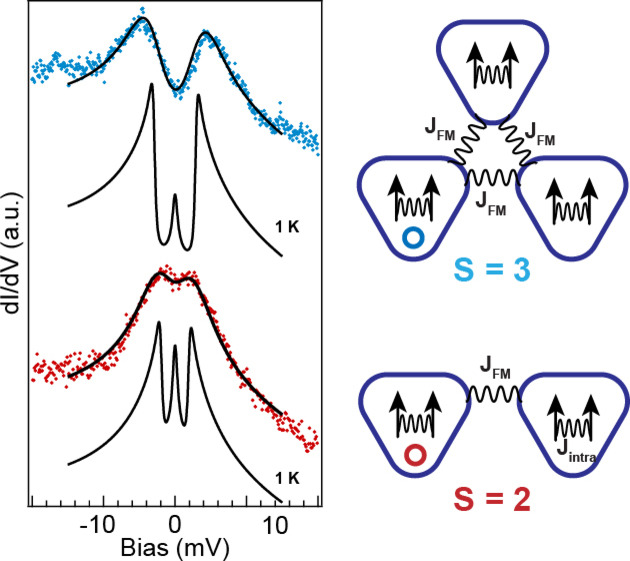

## Introduction

Ferromagnetic exchange is the dominant form of interaction in atomic magnets, where electrons within a given shell tend to maximize the total spin quantum number *S*.^[1]^ It can be regarded as the antithesis of bonding, hence rare to find in organic molecules.^[2]^ The challenge in synthesizing high‐spin organic molecules is to tune the symmetry of the unpaired electrons’ wavefunction, making the parallel spin alignment energetically favorable. The fundamental interest in high‐spin organic molecules and their wide range of prospective applications (spin qubits, optoelectronics and spintronics)[^3^, ^4^, ^5^] boosted tremendous progress in this field, witnessed by the synthesis of stable high‐spin organic molecules, clusters, and polymers.[^6^, ^7^, ^8^, ^9^] The main bottleneck in the synthesis of these materials is the subtle balance between high stability and strong spin‐spin interactions. Despite the advantage of kinetic stabilization for synthesizing persistent radicals in solution, the inclusion of bulky substituents reduces the intermolecular spin‐spin interactions and, consequently, the effective magnetic coupling.

In this regard, the synthesis and stabilization of openshell molecules on metal surfaces through on‐surface synthesis under ultrahigh vacuum conditions offers the possibility of achieving strong intermolecular exchange correlations through covalent coupling of the spin units.[^10^, ^11^, ^12^] Taking advantage of the bipartite honeycomb lattice of graphene, it is possible to design nanographenes (NGs) hosting unpaired *π*‐electrons,^[13]^ with a total spin *S* given by Ovchinnikov's counting rule $S=\left|N_{A}-N_{B}\right|/2$ , where *N_A_* and *N_B_* are the number of carbon atoms in the two sublattices.[^14^, ^15^]

A prominent example is the family of zigzag‐edged triangular NGs, commonly denoted as [n]triangulenes, where *n* indicates the number of benzene rings along one edge.^[16]^ Their total spin *S*=(*n–*1)/2 derives from [*n*–1] zero‐energy modes living on the majority sublattice. This leads to a large Coulomb repulsion and therefore strong intratriangulene ferromagnetic exchange (*J_FM_* >100 meV).^[17]^

**Figure 1:**
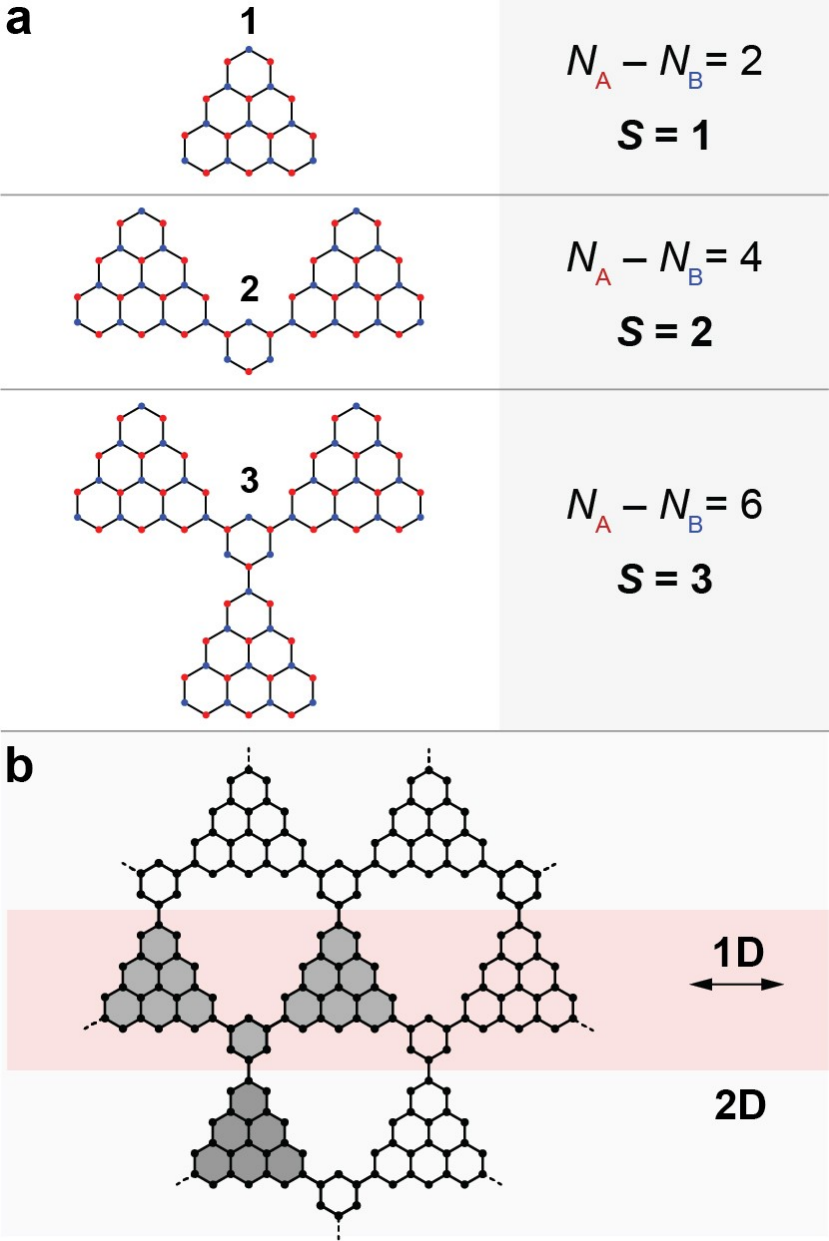
Ferromagnetic coupling of triangulene building blocks. a) Coupling two and three triangulene units via 1,3‐ and 1,3,5‐phenylene spacers leads to structures **2** and **3**, respectively. Ovchinnikov's counting rule predicts a total spin quantum number *S* according to the sublattice imbalance (red and blue filled circles denote the two sublattices). b) Structures **2** and **3** can be regarded as prototypical high‐spin building blocks towards the fabrication of 1D and 2D ferromagnetic materials.

The S=1 triangulene (**1** in Figure 1a) has been the subject of several experimental studies, focusing mainly on its fundamental magnetic properties[^18^, ^19^] and the correlated magnetic ground states emerging from antiferromagnetic coupling of the S=1 building blocks.[^20^, ^21^, ^22^, ^23^, ^24^]

In contrast to the antiferromagnetic case, only a few examples of ferromagnetically coupled NGs have been reported so far.[^25^, ^26^, ^27^, ^28^, ^29^] Furthermore, an in‐depth understanding of the magnetic excitations of degenerate ground states is still lacking. With this aim, we examine the ferromagnetic coupling of two and three S=1 triangulenes, here realized with the corresponding NG structures **2** and **3** (Figure 1a). They can be regarded as prototypical spin clusters, conceived to characterize the relevant spin Hamiltonian parameters and eventually employ **2** and **3** as building blocks for ferromagnetic 1D chains and 2D networks (Figure 1b).

**Scheme 1:**
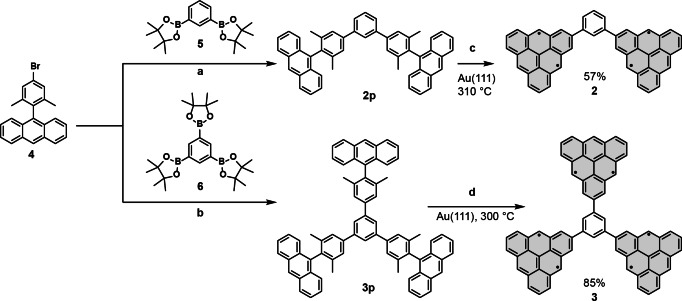
Synthetic route to ferromagnetic triangulene dimer **2** and trimer **3**. Reagents and conditions: (a) PdCl_2_(dppf)CH_2_Cl_2_, K_3_PO_4_, dioxane, 100 °C, 16 h, 34 %. (b) PdCl_2_(dppf)CH_2_Cl_2_, K_3_PO_4_, dioxane, 85 °C, 16 h, 65 %. (c,d) respectively Au (111) held at 310 °C and 300 °C.

The synthetic approach used here consists of an in‐solution synthesis of the precursor **2p** and **3p (see Scheme 1)** and on‐surface synthesis of **2** and **3** upon thermal annealing on a Au(111) surface. By combining STS, STM and theoretical calculations, we then elucidated the spectroscopic features of **2**’s and **3**’s high‐spin ground states.

## Results And Discussion

Triangulene dimer **2** and triangulene trimer **3** were synthesized via a combined in‐solution and on‐surface synthesis approach (see Scheme 1). First, the key building block 9‐(4‐bromo‐2,6‐dimethylphenyl)anthracene **4** was synthesized by a five‐step procedure according to our previous work.^[20]^ Then, Suzuki‐coupling of **4** with commercially available 1,3‐bis(4,4,5,5‐tetramethyl‐1,3,2‐dioxaborolan‐2‐yl)‐benzene **5** or 1,3,5‐tris(4,4,5,5‐tetramethyl‐1,3,2‐dioxaborolan‐2‐yl)benzene **6** gave precursor **2p** and **3p** in 34 % yield and 65 % yield, respectively.

**Figure 2:**
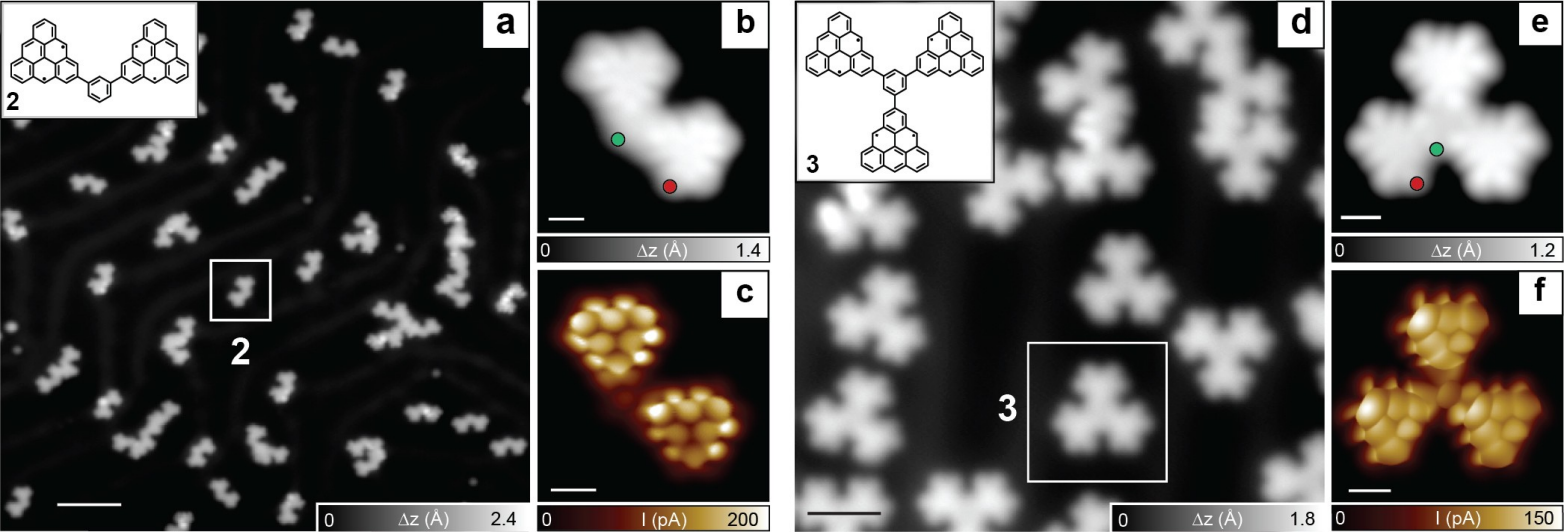
On‐surface synthesis and structural characterization of structures **2** and **3**. (a,d) Overview STM images after the deposition and annealing step of **2p** and **3p** on Au(111) (*V*=−0.1 V, *I*=100 pA). Target structures **2** and **3** are highlighted with squares. The corresponding STM images (b,e) (*V* =−0.6 V, *I* =100 pA) and bond‐resolved STM images (c,f) (acquired with a carbon monoxide functionalized tip and tunneling parameters: *V*=−5 mV, *I* =50 pA, Δ*h*=−0.6 Å) demonstrate the successful synthesis of **2** and **3**. Scale bars: 5 nm (a), 0.5 nm (b,c,e,f) and 2 nm (d).

The target compounds **2** and **3** in Scheme 1 were achieved by on‐surface synthesis starting from molecular precursors **2p** and **3p**, respectively. For the synthesis of **2**, a submonolayer of **2p** was deposited onto an atomically clean Au(111) surface at room temperature (see SI Figure S4a) and subsequently annealed at 310 °C to promote oxidative ring closure of the methyl groups.^[20]^ STM imaging in Figure 2a reveals the presence of a majority of isolated dimers along with covalently bonded molecular clusters. By an accurate inspection of various STM images, we found that 57 % of the isolated dimers show a uniform and symmetric topography consistent with **2** (Figure 2b). Bond‐resolved (BR) STM imaging (Figure 2c) further corroborates this assignment. Similarly, **3** is obtained from the precursor **3p** deposited onto a clean Au(111) surface held at room temperature (Figure S4b) or at 300 °C (Figure 2d). The latter preparation yielded mainly covalently bonded molecular clusters, but 85 % of the isolated molecules feature a uniform clover‐like topography shown in Figure 2e characteristic of structure **3** and demonstrated by BR‐STM imaging in Figure 2f.

### Electronic Structure

**Figure 3:**
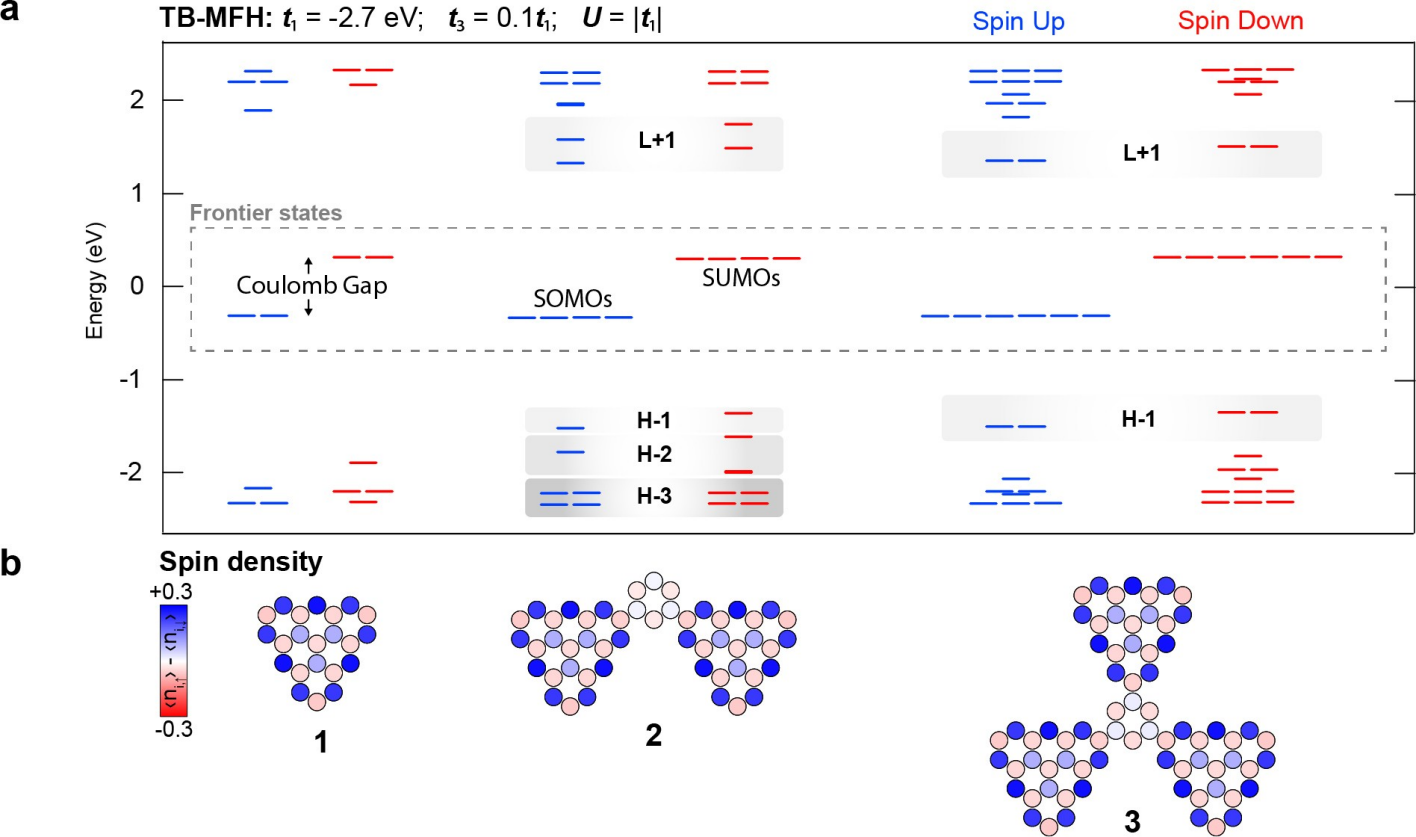
TB‐MFH‐simulated electronic and magnetic properties of **1**, **2** and **3**. (a) Energy spectra calculated using third‐neighbor hopping *t*_3_=0.1*t*_1_ and on‐site Coulomb repulsion *U*=*|t*_1_ *|*. (b) Corresponding spin density plots, where blue and red filled circles denote the populations of spin‐up and spin‐down electrons.

To establish the basic theoretical concepts relevant to the electronic and magnetic properties of the triangulene‐based nanographenes, we first resort to a tight‐binding (TB) level of theory, where electron correlations are considered through the Mean Field Hubbard (MFH‐TB) approximation. Figure 3a shows the energy diagram calculated with first and third neighbor hopping TB‐MFH model for the [3]triangulene monomer (**1**), the ferro dimer (**2**), and the ferro trimer (**3**). As follows from the sublattice imbalance, each triangulene unit hosts two zero energy states (ZES), mainly residing at the zigzag edge and on the same sublattice of the NG,[^30^, ^31^, ^32^] which gives rise to a strong intra‐triangulene Hund exchange. Once the on‐site Coulomb repulsion is considered, these states split into singly occupied (unoccupied) molecular orbitals SOMOs (SUMOs) separated in energy by a Coulomb gap. When connecting the triangulene units through the meta positions of a benzene ring, such as with 1,3‐ or 1,3,5‐ phenylene spacers, the four or six ZES live on the same sublattice, and if we consider a third neighbor hopping $t_{3}\neq 0$ , Hund exchange provides the expected inter‐triangulene ferromagnetic coupling.^[33]^ Accordingly, **2** and **3** are expected to host a S=2 and S=3 ground state, respectively, with the expected spin distributions reported in Figure 3b.

**Figure 4:**
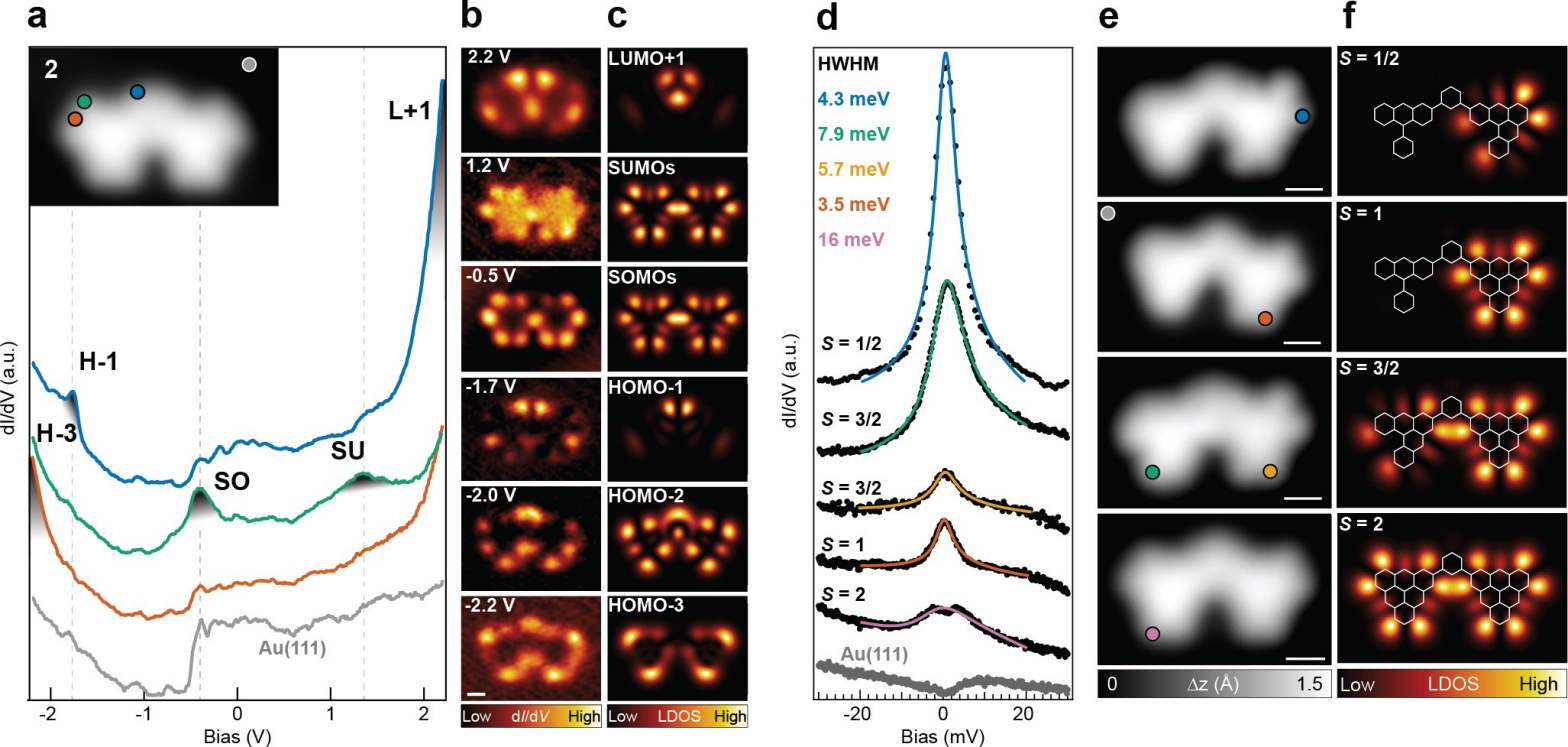
Electronic and magnetic characterization of **2**. (a) d*I*/d*V* spectra acquired with a metal tip at three different locations (marked with filled circles) on **2**, revealing five distinct molecular orbital resonances (MO) (open feedback parameters: *V* =−2.2 V, *I* =350 pA; *V_rms_* =14 mV). (b) Constant‐current d*I*/d*V* spatial maps of the detected MO resonances ( *I*=350 pA; *V_rms_* =14 mV), along with the corresponding MFH‐LDOS maps (c). (d) Low‐bias d*I*/d*V* spectra of **2** and its dihydro‐intermediates (*V_rms_* ≤1.4 mV), achieved through a tip‐induced step‐by‐step dehydrogenation of the very same molecule. The HWHM of each zero‐bias resonance was extracted by Frota fitting^[34]^ and the values are reported in the inset. At each manipulation step, the corresponding low‐bias ( *V*=−0.1 V, *I* =50–100 pA) STM image (e) is compared to the MFH‐LDOS map (f) of the spin‐carrying orbitals of the intermediate structures. Scale bars: 0.5 nm (e).

We now focus on the hitherto unexplored electronic properties of structure **2** on Au(111), while the electronic characterization of **3** is reported in the Supplemental Material.^[35]^ In Figure 4a the STS measurements of an isolated **2** molecule on Au (111) are shown. The differential conductance d*I*/d*V* spectra reveal the presence of five distinct resonances located at different positions on the molecule. To investigate how these resonances of the local density of states (LDOS) extend over the molecule, we carried out constant‐current d*I*/d*V* maps at each resonance energy (Figure 4b). A direct comparison with the calculated TB‐MFH LDOS maps (shown in Figure 4c) allows proper labeling of the experimentally observed resonances. As expected, the frontier orbitals are singly occupied (unoccupied) electronic states sharing the very same LDOS, separated in energy by a Coulomb gap of 1.7 eV. The latter argument, bolstered by the perfect match of the calculated LDOS with the experimental resonances, validates our initial assumption on the S=2 ground state of **2**.

### Magnetic Characterization

We now shift our focus to the low‐energy manifestations of **2**’s magnetic ground state. The coupling design via *meta*‐junctions is predicted to lead to weak ferromagnetic interactions, based on a combination of ferromagnetic Hund exchange (via *t*_3_) and Coulomb‐driven superexchange mechanisms.^[33]^ Therefore, two features are expected in a differential conductance spectrum. On the one hand, inelastic spin‐spin scattering will lead to steps symmetric in energy around zero bias voltage,^[36]^ similar to the observation of antiferromagnetically coupled triangulenes.^[20]^ On the other hand, the *S*=2 degenerate ground state of **2** allows for elastic spin‐scattering, which can be observed as a characteristic zero‐bias Kondo resonance.^[37]^ To better resolve these low‐energy properties, we map the low‐bias d*I*/d*V* spectrum as a function of the total spin *S* using step‐by‐step tip‐induced dehydrogenation of a dimer molecule where the four spins were initially absent due to hydrogen passivation (more details in SI^[35]^). Figures 4d,e show respectively the d*I*/d*V* spectrum and the STM image after each manipulation step. The correct assignment of the ground state *S* to the respective chemical structure is corroborated by the calculated MFH‐LDOS maps of the frontier MOs (Figure 4f), where the presence of a dihydro group is taken into account in the MFH model by removing the corresponding carbon site from the π‐electron system.[^38^, ^39^] Both $S=\frac{1}{2}$ and S=1 intermediates feature a Kondo peak, albeit with distinct relative amplitudes. This observation is consistent with previous findings.[^18^, ^40^, ^41^] The asymmetric spin system spin$\frac{1}{2}$ ‐spin1 ($S=\frac{3}{2}$ ) shows a significantly wider resonance compared to cases $S=\frac{1}{2}$ and S=1 . This observation may indicate the coexistence of low‐energy magnetic excitations and a zero‐energy peak; however, these energy scales are challenging to resolve at the experimental temperature of 4.5 K and therefore subject to future analysis. To quantitatively evaluate these differences, the half width at half maximum (HWHM) of each zero bias resonance was extracted by fitting the measured lineshape with a Frota function^[34]^ and the corresponding values are reported in the inset of Figure 4d. Unlike the dimer intermediates, it is evident that the S=2 d*I*/d*V* spectrum cannot be fitted with a single Frota function, therefore requiring a more accurate analysis of its magnetic properties, as described below.

**Figure 5:**
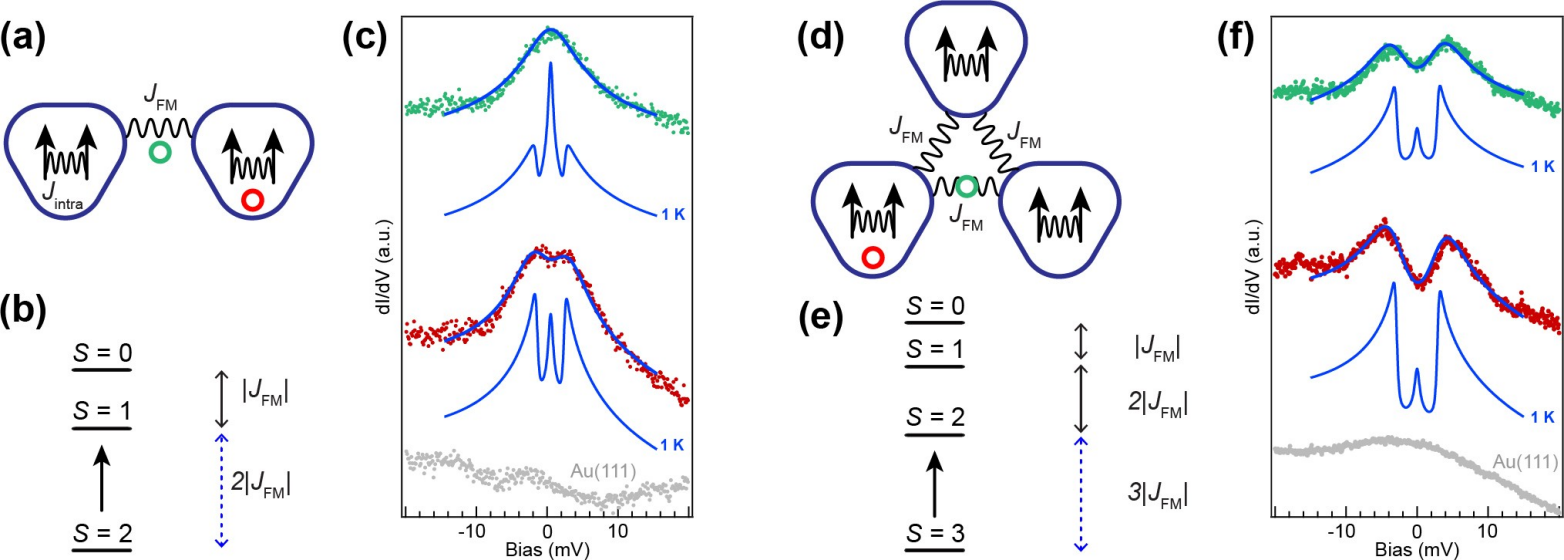
Comparison of the magnetic properties of **2** and **3**. (a,d) Heisenberg models for **2** and **3**, where each triangulene is considered as a *S*=1 unit. (b,e) Corresponding energy level scheme obtained by analytical solution of the Heisenberg model and confirmed by CAS calculations. (c,f) High‐resolution d*I*/d*V* spectroscopy acquired respectively on **2** and **3** on two distinct locations for each molecule, depicted as coloured filled circles in Figure 2 (b,e). By including spin‐spin scattering processes (perturbative part) into the d*I*/d*V* formalism and solving to the third order, the measured spectra can be nicely fitted and the ferromagnetic exchange extracted, which is found to be approximately 1 meV for both **2** and **3**. Open feedback parameters d*I*/d*V* spectra: (c) *V*=−30 mV, *I*=600 pA; (f) *V*=−20 mV, *I* =500 pA. Lock‐in modulation (c) *V_rms_* =350 *μ*V, (f) *V_rms_* =700 *μ*V.

Figure 5c shows high‐resolution d*I*/d*V* spectra highlighting the low‐energy magnetic excitations measured at two distinct locations on **2** with a metal tip. The qualitative difference of the spectra acquired at the two locations on the molecule is evident. Although the spectrum acquired on the central part of the molecule (green spectrum) appears to be a broad zero‐bias peak, the one taken on the outer side of the triangulene unit (red spectrum) clearly reveals the presence of two peaks at about ±2 meV. As shown in Figure S5,^[35]^ very similar low‐energy features are inherently present in the STS spectrum if CO‐functionalized tips are used^[42]^ (because of inelastic vibrational excitations); therefore, to avoid erroneous interpretations, only data acquired with metal tips will be considered.

To shed light on these elusive magnetic features and deepen our understanding of **2**, we first resort to theory by considering the six and eight TB single particle states closest to the Fermi level and solving the many‐body Hubbard Hamiltonian exactly in this restricted basis, which is commonly known as CAS(6,6) and CAS(8,8) calculations (reported in SI^[35]^), respectively. We find the outcome of both calculations to be in line with the expected magnetic spectrum, with the triplet and singlet excited states, respectively, $2\left|J_{FM}\right|$ and $3\left|J_{FM}\right|$ away from the quintet ground state (excitation spectrum in Figure 5b). On this basis, we can now represent **2** with a simple Heisenberg Hamiltonian (Figure 5a) where two spin‐1 units $\vec{S_{1}}$ and $\vec{S_{2}}$ are coupled by an effective ferromagnetic interaction $J_{FM}\vec{S_{1}}\cdot \vec{S_{2}}$ . By including a tunnel junction and the corresponding spin‐flip scattering processes up to the third order, we obtain the calculated d*I*/d*V* intensity^[43]^ (here depicted by the blue spectra). By fitting the d*I*/d*V* spectrum in red (Figure 5c), a value of $\left|J_{FM}\right|$ of 0.98 meV is obtained (more details on the fitting procedure can be found in the Supplemental Material^[35]^). To better visualize the inherent magnetic excitations, we recalculate the resulting fit at an effective temperature of 1K and report the results below. The latter reveals two distinct features: a sharp zero energy resonance—the result of third‐order spin scattering processes of the degenerate ground state—and two symmetric peaks at about ±2 meV due to the spin‐flip transition between the S=2 ground state and the S=1 excited state (allowed by spin selection rules). The spatial extension of the quintet to triplet spin excitation is reported in Figure S6.^[35]^ If we now fix $\left|J_{FM}\right|$ and try to fit the green spectrum acquired in the center of **2**, the fit does not converge. Indeed, when the tip is placed between the two spin units, third‐order spin scattering processes can occur simultaneously on both units, which is reflected by an increased zero‐energy peak with respect to the spin excitation peaks, as seen in the calculated 1 K spectrum. By including this effect, we obtain a perfect match with the experimental data, while keeping the very same Kondo scattering term $J_{\rho }$ and the same effective temperature *T_eff_*.

With the elucidation of the mechanisms underlying **2**’s low‐energy magnetic excitations, we are now able to extend the analysis to **3** and compare the two systems. The computationally challenging CAS(10,10) corroborates the ferromagnetic solution for **3**, with three excited states defined by spin quantum numbers S=2 , S=1 and S=0 separated in energy from the septet (S=3 ) ground state by $3\left|J_{FM}\right|$ , $5\left|J_{FM}\right|$ and $6\left|J_{FM}\right|$ , respectively, as depicted in the energy scheme in Figure 5e . The d*I*/d*V* spectra measured on **3** and reported in Figure 5f reveal the presence of low‐energy magnetic excitations ascribed to the septet‐quintet transition, while transitions from the ground state to higher‐energy states are forbidden by spin selection rules. Since the first excited state consists of two degenerate quintet states, the relative zero‐bias peak intensity is expected to appear smaller compared to **2** with a single triplet state as excited level. In addition, the exponential reduction of the Kondo temperature T_K_ with the total spin *S* ^[40]^ results in a significantly reduced Kondo scattering term *J*_*ρ*_ for the *S*=3 with respect to the *S*=2 system. To fit the experimental data and extract $\left|J_{FM}\right|$ , we resort to the previously adopted Heisenberg model (Figure 5d). Using the very same scattering parameters and $\left|J_{FM}\right|$ adopted in **2** an excellent fit of the red spectrum in Figure 5f is obtained. By placing the tip in between two spin‐1 units, an increased intensity of the zero‐energy peak is recorded, due to the simultaneous scattering of the two distinct spin units.

The in‐depth analysis of **2** and **3** carried out in this work highlights the relevance of high‐order spin scattering effects in the low‐energy magnetic behavior of systems with degenerate ground states. We have demonstrated the accuracy of our approach by successfully extending **2**’s Hamiltonian parameter space to the more complicated S=3 system (labeled **3**), thus widening the future implications of our work to extended ferromagnetically coupled spin systems.

## Conclusions

We have reported the successful on‐surface synthesis of high‐spin NGs where triangulene spin‐1 units are ferromagnetically coupled via 1,3‐ and 1,3,5‐phenylene spacers into dimers and trimers. The considered bonding scheme is predicted to create magnetic NGs with a total spin quantum number *S* equal to the number of connected triangulene units. By high‐resolution STS we have carried out a comprehensive electronic characterization of both systems, revealing the presence of low‐energy magnetic excitations. By comparing our results with different levels of theory, we have determined an open‐shell S=2 and S=3 ground state for the dimer and trimer, respectively, ascribing the observed low‐energy features to quintet‐triplet and septet‐quintet spin excitations. Despite the relatively weak ferromagnetic exchange coupling intrinsic to the employed bonding scheme, our analysis highlights the important role of quantum correlations embodied by the observed zero‐energy peaks.

## Conflict of Interests

The authors declare no conflict of interest.

## Supporting information

As a service to our authors and readers, this journal provides supporting information supplied by the authors. Such materials are peer reviewed and may be re‐organized for online delivery, but are not copy‐edited or typeset. Technical support issues arising from supporting information (other than missing files) should be addressed to the authors.

Supporting Information
