# Supplementary material for: Magnetic Excitations in Ferromagnetically Coupled Spin‐1 Nanographenes
Source: Angew Chem Int Ed Engl. 2024 Nov 6;63(52):e202412353. doi: 10.1002/anie.202412353 (PMC11656132; doi:10.1002/anie.202412353)
Supplement: Supplementary file 1 — Supporting Information [file ANIE-63-e202412353-s001.pdf]

Angewandte Chemie
Eine Zeitschrift der Gesellschaft Deutscher Chemiker

Supporting Information

Magnetic Excitations in Ferromagnetically Coupled Spin-1 Nanographenes

E. Turco, F. Wu, G. Catarina, N. Krane, J. Ma, R. Fasel, X. Feng*, P. Ruffieux**

Supplemental Material for: Magnetic Excitations in Ferromagnetically Coupled Spin-1 Nanographenes

Elia Turco,[+,*,a] Fupeng Wu,[+,b] Gonçalo Catarina,[a] Nils Krane,[a] Ji Ma,[b]
Roman Fasel,[a,c] Xinliang Feng,[*,b] Pascal Ruffieux[*,a]

1. Experimental Methods

1.1. Sample Preparation and Scanning Probe Measurements

STM measurements were performed with a commercial low-temperature STM/AFM from Scienta Omicron operated at a temperature of 4.5 K and a base pressure below $5 \cdot 10^{-11}$ mbar. The Au(111) single crystal surfaces were prepared by iterative Ar^{+} sputtering and annealing cycles. Before sublimation of molecules, the surface quality was verified through STM imaging. The powders of **2p** and **3p** precursors were filled into quartz crucibles of a home-built evaporator and sublimed at 280 ° C and 290 ° C, respectively, on the surfaces of the single crystal. STM images were acquired in both constant-current (overview and high-resolution imaging) and constant-height (bond-resolved imaging) modes, $\mathrm{d}I/\mathrm{d}V$ spectra were acquired in constant-height mode, and $\mathrm{d}I/\mathrm{d}V$ maps were acquired in constant-current mode. Indicated bias voltages are given with respect to the sample. Unless otherwise noted, all measurements were performed with metallic tips. Differential conductance $\mathrm{d}I/\mathrm{d}V$ spectra and maps were obtained with a lock-in amplifier. Modulation voltages (root mean square amplitude V_{rms}) for each measurement are provided in the respective figure caption. Bond-resolved STM images were acquired in constant-height mode with CO-functionalized tips at low bias voltages while recording the current signal. Open feedback parameters on the molecular species and the subsequent lowering of the tip height (Δz) for each image are provided in the respective figure captions. The data was processed with Wavemetrics Igor Pro software.

1.2. Hydrogen passivation

In figure 4 (d,e) of the main text, dihydro intermediates of **2** are reported. These structures are formed by passivation of **2**'s *active* spin sites by hydrogen diffusion on the metal surface, subsequently to the cyclodehydrogenation reaction step. Therefore, it naturally occurs to find structures where the unpaired electrons are partially or fully quenched by dihydro groups, thus allowing the detection and sequential manipulation of intermediates with various spin ground states S. More details on the tip-based manipulation method can be found in Ref. [1].

1.3. Yield of the target compounds

The reported **2's** and **3's** yields were evaluated by assessing the percentage of target molecules (with flat and uniform topography) compared to the total number of single molecules. For each system, various overview STM images were analyzed and the statistics were computed on a sample larger than a hundred of molecules. The electronic and magnetic characterization reported in the manuscript was carried out exclusively on target molecules that were found to be stably adsorbed on a flat face-centered cubic (*fcc*) region of the herringbone reconstruction. Molecules too close to the elbows of herringbone ridges were avoided because they are known to be reactive sites because of lower atomic coordination.

2. Computational Methods

2.1. Tight-binding and mean-field Hubbard calculations

TB-MFH calculations were performed by numerically solving the mean-field Hubbard Hamiltonian with third-nearest-neighbor hopping.

The corresponding Hamiltonian reads as

$$\hat{\mathcal{H}}_{\mathrm{MFH}} = \sum_{j} \sum_{\langle \alpha,\beta \rangle j,\sigma} t_j \hat{c}^{\dagger}_{\alpha,\sigma} \hat{c}_{\beta,\sigma} + U \sum_{\alpha,\sigma} \langle n_{\alpha,\sigma} \rangle n_{\alpha,\bar{\sigma}} - U \sum_{\alpha} \langle n_{\alpha,\uparrow} \rangle \langle n_{\alpha,\downarrow} \rangle, \tag{1}$$

Here, ${c_{\alpha,\sigma}}^{\dagger}$ and c_{β}, σ denote the spin selective ($\sigma \in \uparrow, \downarrow$) creation and annihilation operator at sites α and β, $\langle \alpha, \beta \rangle_j$ ($j = 1, 3$) denotes the nearest-neighbor and third-nearest-neighbor sites for j = 1, and 3, respectively, t_j denotes the corresponding hopping parameters (with $t_1 = 2.7$ eV and $t_3 = 0.1 t_1$ for nearest-neighbor and third-nearest-neighbor hopping), U denotes the on-site Coulomb repulsion, $n_{\alpha,\sigma}$ denotes the number operator, and $\langle n_{\alpha,\sigma} \rangle$ denotes the mean occupation number at site α. Orbital electron densities, ρ, of the nth-eigenstate with energy E_n have been simulated from the corresponding state vector $a_{n,i,\sigma}$ by

$$\rho_{n,\sigma}(\vec{r}) = \left| \sum_{i} a_{n,i,\sigma} \phi_{2p_z}(\vec{r} - \vec{r_i}) \right|^2, \tag{2}$$

where i denotes the atomic site index and ϕ_{2p_z} denotes the Slater $2p_z$ orbital for carbon. All TB-MFH calculations presented in the manuscript were done in the third-nearest-neighbor approximation and using an on-site Coulomb term $U = |t_1|$.

2.2. Calculation of differential conductance spectra

First, the spin hamiltonian is constructed by considering each triangulene as a spin-1 unit and a ferromagnetic Heisenberg-like exchange J_{inter} between neighboring units, while the magnetic anysotropy term and non-collinear Dzyaloshinskii–Moriya spin coupling are irrelevant for this system. Notably, if we instead consider each unit as two ferromagnetically coupled spins with $J_{intra} \gg J_{inter}$, the result is identical. The $\mathrm{d}I/\mathrm{d}V$ spectra were simulated by introducing a perturbative term into the spin Hamiltonian, which accounts for spin-flip processes up to third order in the interaction matrix elements [2].

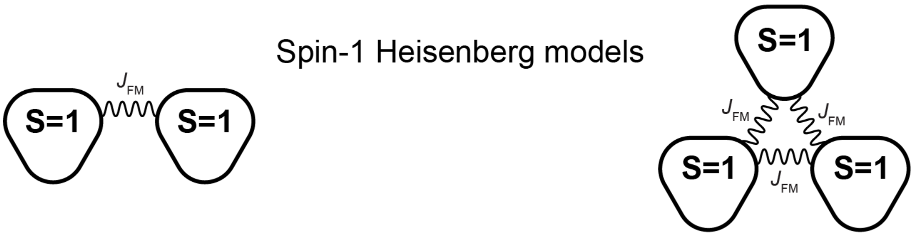

Figure S1. Heisenberg model of the dimer **2** and trimer **3**.

The data shown in Fig.4c of the manuscript have been fitted using the fitting procedures provided in ref. [2]. In the following, I will briefly describe the fitting *modus operandi*.

Frozen parameters

- $\omega_0 = 20$ (The bandwidth of the scattering substrate electrons in meV.
- $U = 0$ (The Coulomb scattering parameter)
- $\eta = 0$ (The spin polarization of the tip)
- $V_{off} = 0$ (Voltage offset in mV)

Fitting parameters

- J_{FM} (Ferromagnetic Heisenberg coupling strength)
- $J\rho_s$ (The Kondo scattering parameter).
- T_0^2 (Tunnel barrier coupling constant, as scaling factor)
- T_K (Effective temperature in Kelvin)
- b, σ_0 (respectively sloped and conductance offset)

Note: First the the fitting parameters were manually set to get close to the observed spectrum, then the fitting procedure was initiated. In a second step, to accurately determine J_{FM}, the fitting was repeated but keeping some of the fitting parameters (b, σ_0, T_0^2) frozen.

The spectra acquired in the external part of **2** was employed as model system to define the parameter space. To reproduce the spectrum taken in the central part of the molecule, third order scattering processes through both spin centers were considered. The fit was carried out keeping the same $J_{FM} = 0.98$meV.

Table 1. Parameters used to fit / simulate the experimental $\mathrm{d}I/\mathrm{d}V$ spectra reported in Figure 5. Spectra in Fig. 5(c) were fitted, while for the spectra in (f), fitting was not possible and a fine-tuning approach was employed.

	J_{FM}	$J\rho_s$	T_0^2	b	V_{off}	σ_0	T_{eff}
(c) red	0.98	0.19	0.046	-0.005	0.5	2.99	7.88
(c) green	0.98	0.25	0.024	-0.0002	0.5	7.03	7.88
(f) red	0.98	0.19	0.12	-0.006	0.0	-1.37	7.88
(f) green	0.98	0.31	0.055	-0.002	0.0	2.3	7.88

2.3. CAS calculations

The starting point for the CAS calculations is a tight-binding model where we only consider the p_z orbitals of the carbon atoms that compose the nanographenes. We consider a typical value for the first neighbor hopping, $t_1 = -2.7$ eV. Moreover, we take a third neighbor hopping $t_3 = 0.1t_1$, in line with previous work and essential to capture the magnetic properties of triangulene lattices [3]. The corresponding Hamiltonian reads as

$$\hat{\mathcal{H}}_0 = t_1 \sum_{\sigma} \sum_{\langle i,j \rangle} \hat{c}^{\dagger}_{i,\sigma} \hat{c}_{j,\sigma} + t_3 \sum_{\sigma} \sum_{\langle i,j \rangle} \hat{c}^{\dagger}_{i,\sigma} \hat{c}_{j,\sigma}, \tag{3}$$

where $\hat{c}_{i,\sigma}$ denotes the annihilation operator for an electron in carbon site i with spin $\sigma = \uparrow, \downarrow$. This single-particle Hamiltonian can be easily diagonalized numerically, leading to a set of molecular orbitals. The Fermi level μ is defined assuming nanographenes at charge neutrality, i.e., one electron per carbon site.

In our CAS calculations, we first choose an active space of molecular orbitals close to the Fermi level. The underlying approximation is to assume that all molecular orbitals with energy below (above) the active space are doubly occupied (empty). Then, we include interactions in the Hubbard form,

$$\hat{\mathcal{H}}_U = U \sum_{i} \hat{n}_{i,\uparrow} \hat{n}_{i,\downarrow}, \tag{4}$$

where $U > 0$ is the on-site Hubbard repulsion, taken as a free parameter, and $\hat{n}_{i,\sigma} = \hat{c}^{\dagger}_{i,\sigma} \hat{c}_{i,\sigma}$. Finally, the total (many-body) Hamiltonian $\hat{\mathcal{H}} = \hat{\mathcal{H}}_0 + \hat{\mathcal{H}}_U$ is represented in a restricted basis set—where we consider all multielectronic configurations that can be obtained with N_e electrons in the previously selected N_{MO} molecular orbitals—and diagonalized numerically. This is referred to as the CAS(N_e,N_{MO}) approximation. Importantly, given that we consider nanographenes described by a bipartite lattice at half filling, $\hat{\mathcal{H}}_0$ features molecular orbitals with symmetric energy with respect to $\mu = 0$. Therefore, our active space contains all (singly-occupied) zero-energy states, plus an equal number of molecular orbitals with energy above and below μ, which implies $N_e = N_{MO}$.

In Fig. S2, we show the (single-particle) tight-binding energy levels of ferromagnetic triangulene dimers and trimers. Given that benzene spacers link triangulenes via the same sublattice, dimers (trimers) have a sublattice imbalance of 4 (6), which implies the existence of the observed 4 (6) zero-energy states [4]. These singly-occupied zero-energy states constitute the minimal active space that can be used in our calculations, namely CAS(4,4) for dimers and CAS(6,6) for trimers. Going beyond the minimal active space—up to CAS(10,10) due to memory constraints—allows to include corrections due to higher-energy molecular orbitals, although convergence of such Coulomb-driven exchange contributions is known to be problematic [5]. Therefore, our CAS calculations do not aim at providing a quantitative description of the experimental results (namely the value of the exchange coupling), but rather to certify the picture of a ferromagnetic spin model.

In Fig. S3a, we show our CAS calculations for the ferromagnetic triangulene dimer, considering the largest affordable active space for this system[1] and taking $U = |t_1|$ as a reference value, usual for nanographenes [1,6]. We observe a low-energy manifold, composed of 9 states, well separated from the remaining higher-energy states.

[1] Note that degeneracies forbid the use of CAS(10,10).

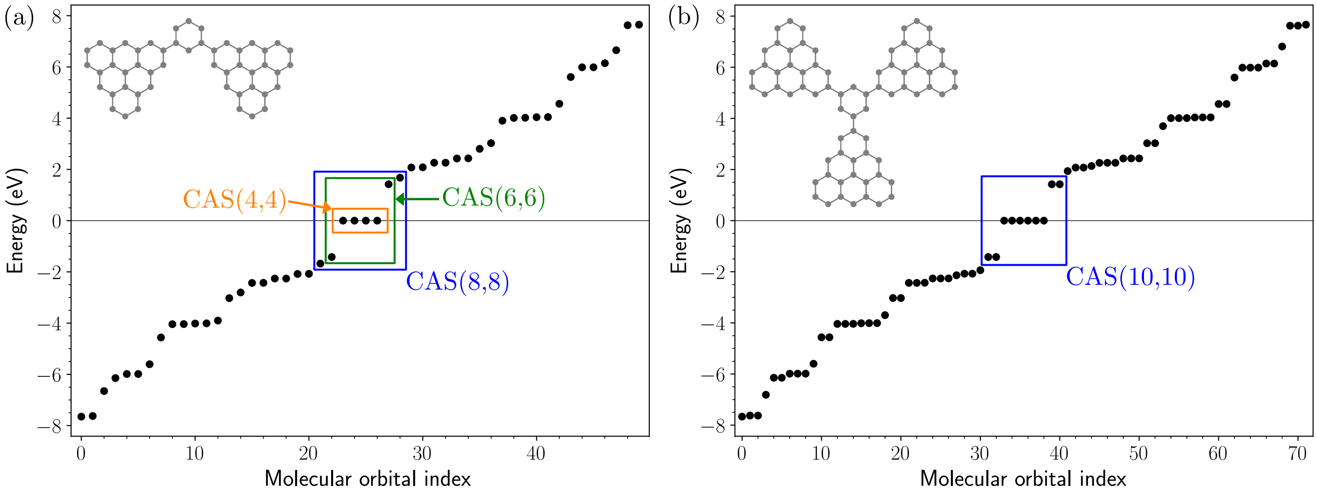

Figure S2. Energy levels, obtained with the tight-binding model, for (a) ferromagnetic triangulene dimer and (b) ferromagnetic triangulene trimer. Solid black lines denote the Fermi level. Boxes represent the different choices of active space used in the CAS calculations of Fig. S3.

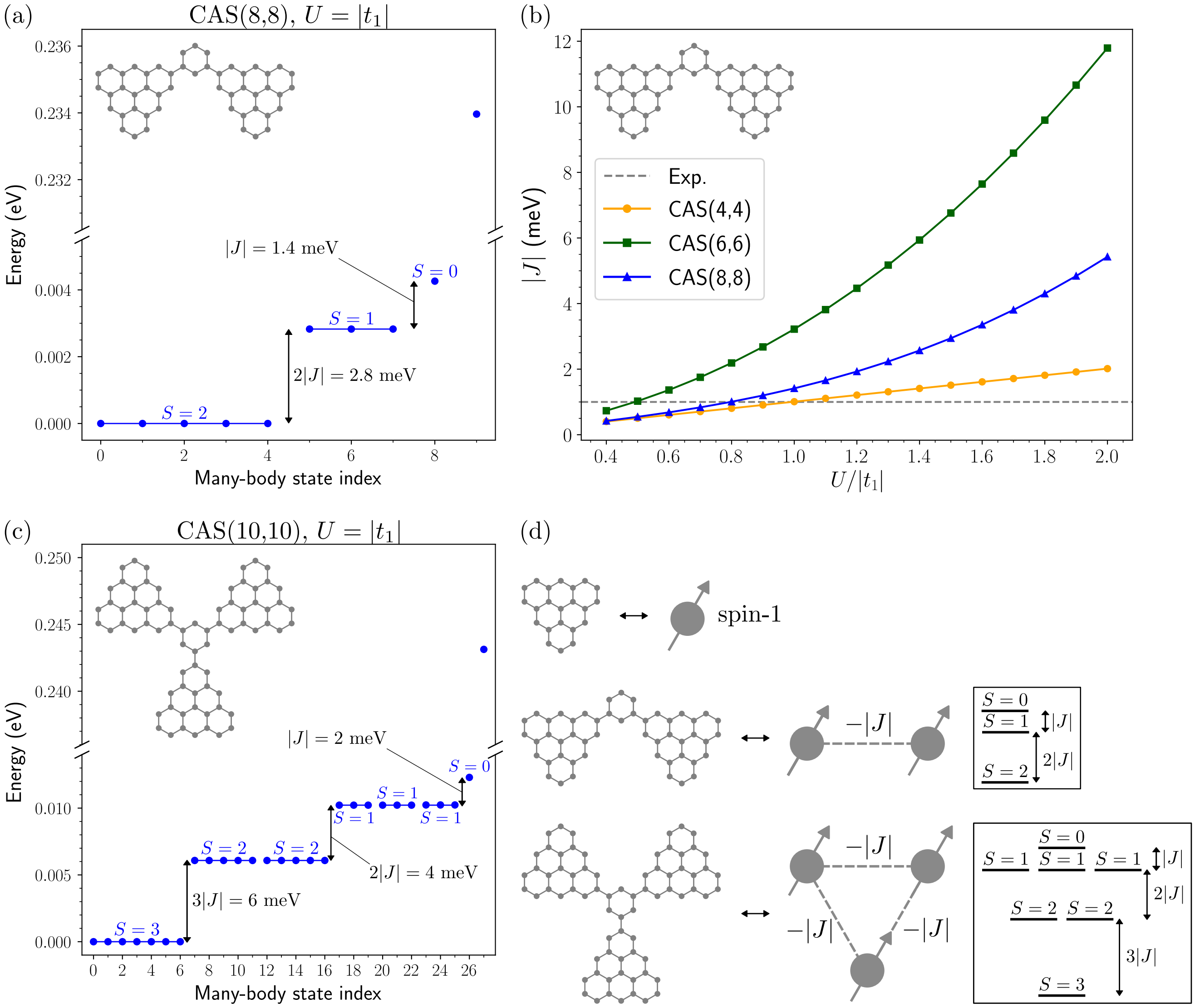

Figure S3. CAS calculations and mapping to the ferromagnetic spin model. (a) Many-body energy levels, obtained with CAS(8,8) and $U = |t_1|$, for the ferromagnetic triangulene dimer. (b) Exchange coupling, obtained as half of the quintet-triplet splitting of the ferromagnetic triangulene dimer, as a function of U, for different choices of active space. The gray dashed line denotes the experimental value. (c) Many-body energy levels, obtained with CAS(10,10) and $U = |t_1|$, for the ferromagnetic triangulene trimer. (d) Scheme illustrating the mapping between triangulenes coupled via same-sublattice benzene spacers and ferromagnetic spin-1 Heisenberg models. The agreement between the energy levels of the spin models, shown in the right panels, and the low energy manifold of the CAS calculations (a,c), validates the use of a ferromagnetic spin-1 Heisenberg Hamiltonian and allows the theoretical determination of the exchange coupling constant $|J|$.

Importantly, this low-energy manifold is compatible with the energy levels of a ferromagnetic spin-1 Heisenberg dimer (Fig. S3d), sharing the same degeneracy pattern ($S = 2$ ground state, followed by $S = 1$ and $S = 0$ excited states) and the same ratio of excitation energies (quintet-triplet excitation energy being twice as large as the triplet-singlet). Analogous conclusions are drawn for the trimer, as shown in Fig. S3c. Therefore, we justify the use of a ferromagnetic spin-1 Heisenberg model to describe these systems. Finally, regarding the magnitude of the exchange coupling constant $|J|$, Fig. S3b shows that, despite significant oscillations with respect to the choice of the active space, our CAS calculations with $U \sim |t_1|$ are in reasonable agreement with experiments, where ferromagnetic exchange couplings of 1 meV were inferred from IETS.

3. Supporting STM, STS and theoretical data

3.1. Precursors 2p and 3p as deposited on Au(111)

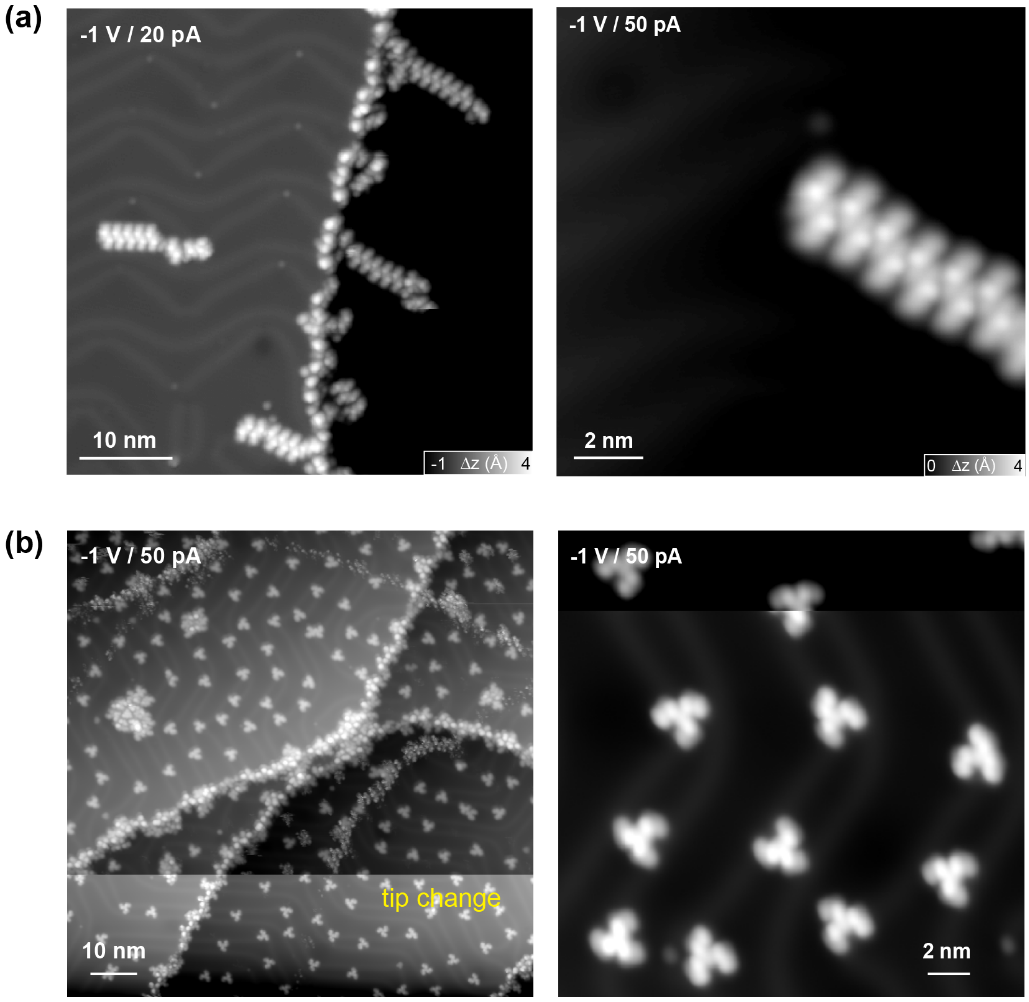

Figure S4. Overview STM images of sub-monolayer coverages of **2p** (a) and **3p** (b) deposited on a Au(111) surface held at room temperature.

3.2. STS with carbon monoxide (CO) functionalized tip

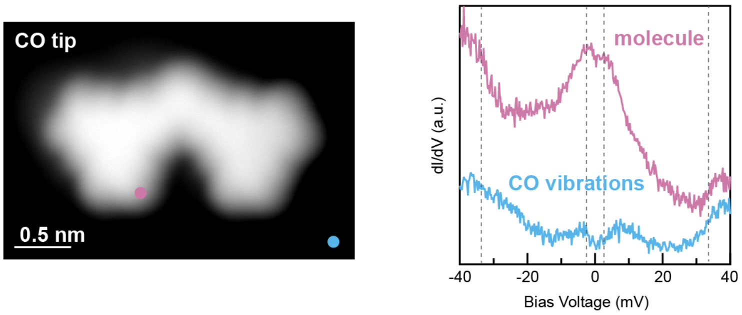

Figure S5. HR-STS spectroscopy with CO-functionalized tip acquired on molecule **2** and on the bare Au(111) surface. The spectra reveal the presence of vibrational inelastic excitations, which can be misinterpreted as spin-excitation steps. Open feedback parameters dI/dV spectra: (c) $V = -50$ mV, $I = 800$ pA. Lock-in modulation $V_{rms} = 700$ μV.

3.3. Mapping spin excitations of 2

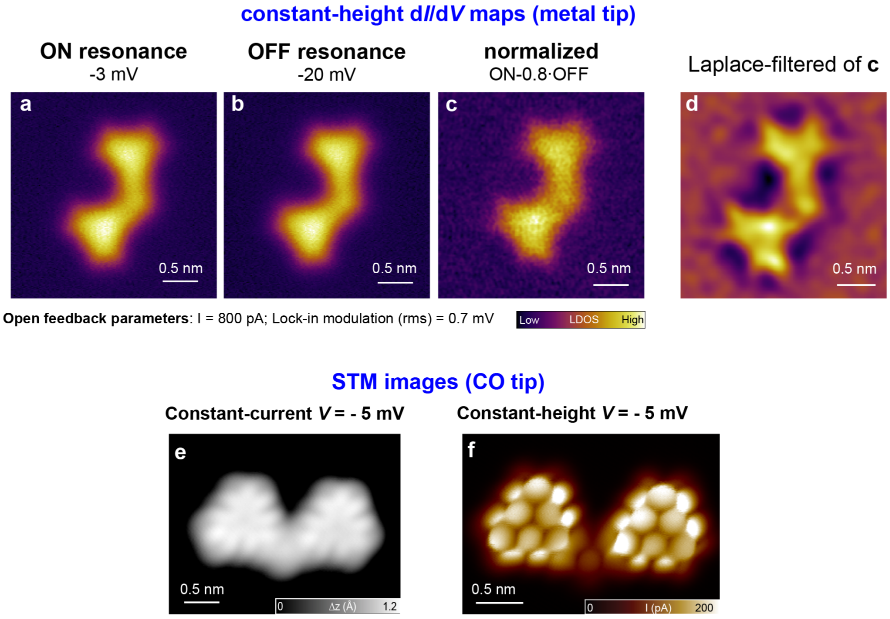

Figure S6. Top: constant-height maps of **2** acquired with a metal tip at the spin excitation threshold (a), off-resonance (b) and the corresponding normalized (subtracted) map (c). On the right (d) is the Laplace-filtered image of the normalized map, highlighting the characteristic features of the frontier orbitals. Open feedback and lock-in parameters are reported in the figure. Bottom: (e) constant-current and (f) constant-height STM imgages acquired with a CO-functionalized tip, revealing the spatial localization of the low-energy excitations. Scanning parameters: (e) $V = -5$ mV, $I = 100$ pA; (f) open feedback $V = -5$ mV, $I = 50$ pA, $\Delta z = -70$ pm.

3.4. Trimer's electronic Characterization

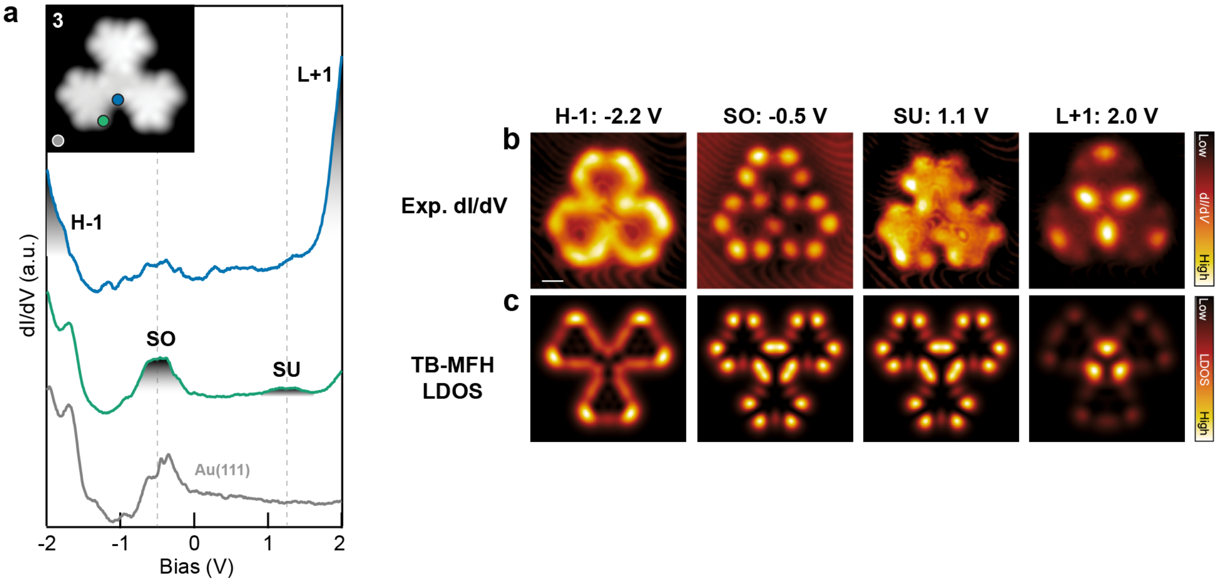

Figure S7. Experimental electronic of **3**. (a) dI/dV spectroscopy acquired with a metal tip on three different locations (marked with filled circles) on molecule **3**, revealing four distinct molecular orbital (MO) resonances (open feedback parameters: $V = -2.0$ V, $I = 350$ pA; $V_{rms} = 18$ mV). (b) Constant-current dI/dV spatial mapping of the detected MO resonances ($I = 300$ pA; $V_{rms} = 19$ mV), along with the corresponding MFH-LDOS maps (c). Notably, the resonances labelled as H-1 and L+1 represent a superposition of higher-order occupied and empty molecular orbitals.

4. Synthetic procedures

4.1. General Methods and Materials

Unless otherwise noted, commercially available starting materials, reagents, catalysts, and dry solvents were used without further purification. Reactions were performed using standard vacuum-line and Schlenk techniques. All starting materials were obtained from TCI, Sigma Aldrich, abcr, Alfa Aesar, Acros Organics, or Fluorochem. The catalysts were purchased from Strem. Column chromatography was performed on silica (SiO_2, particle size 0.063-0.200 mm, purchased from VWR). Silica-coated aluminum sheets with a fluorescence indicator (TLC silica gel 60 F254, purchased from Merck KGaA) were used for thin-layer chromatography. Dichloromethane-d_2 (99.9 atom % D) and 1,1,2,2-Tetrachloroethane-d_2 ($\geq$99.5 atom % D) were purchased from Sigma Aldrich. The key building block 9-(4-bromo-2,6-dimethylphenyl)anthracene (**4**) was synthesized through five-step procedures in our previous work[7].

NMR data were recorded on a Bruker AV-II 300 spectrometer operating at 300 MHz for ^{1}H and 75 MHz for ^{13}C. Measurements were made at room temperature (296 K; AV-II 300) unless otherwise stated. Chemical shifts (δ) are reported in ppm. The coupling constants (J) are reported in Hz. Dichloromethane-d_2 ($\delta(^1\text{H}) = 5.32$ ppm, $\delta(^{13}\text{C}) = 53.8$ ppm) and 1,1,2,2-Tetrachloroethane-d_2 were used as solvent. The following abbreviations are used to describe peak patterns as appropriate: s = singlet, d = doublet, t = triplet, q = quartet, and m = multiplet. High-resolution matrix-assisted laser desorption/ionization time-of-flight (MALDI-TOF) MS was recorded on a Bruker Autoflex Speed MALDI-TOF MS (Bruker Daltonics, Bremen, Germany). All of the samples, were prepared by mixing the analyte and the matrix, 1,8-dihydroxyanthracen-9(10H)-one (dithranol, purchased from Fluka Analytical, purity > 98%) or *trans*-2-[3-(4-tert-butylphenyl)-2-methyl-2-propenylidene]malononitrile (DCTB, purchased from Sigma Aldrich, purity > 99%) in the solid state.

4.2. Detailed Synthetic Procedures

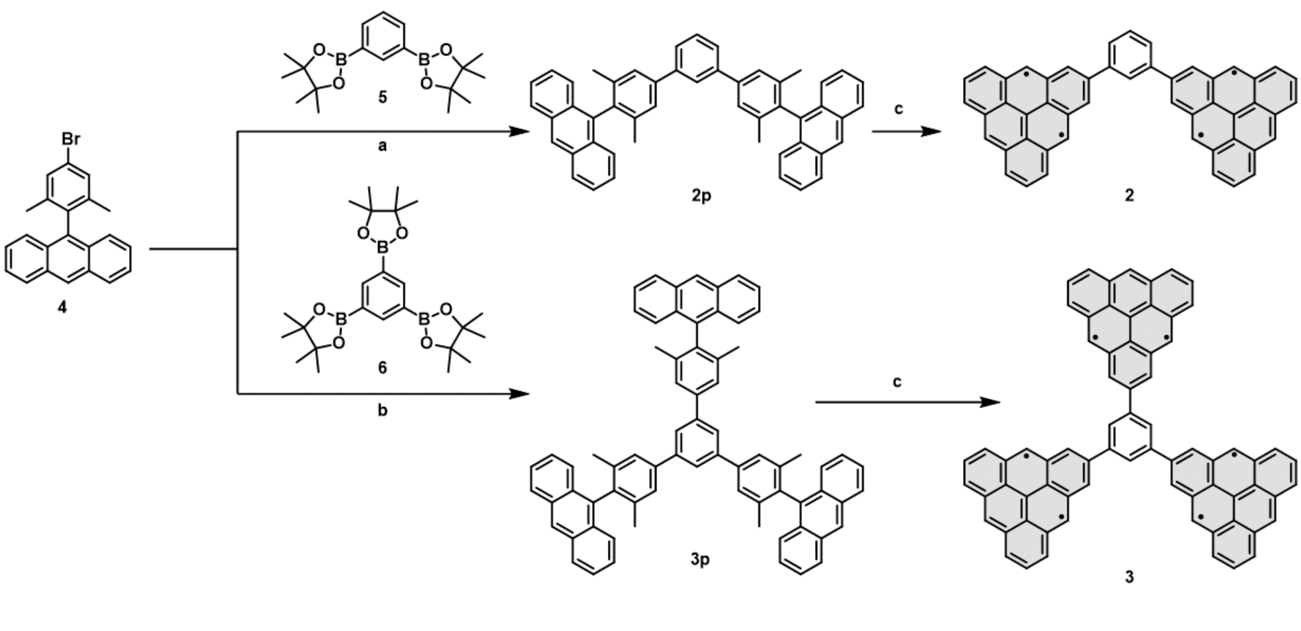

Figure S8. Synthetic route to ferromagnetic triangulene dimer **2** and ferromagnetic triangulene trimer **3**. Reagents and conditions: (a) $PdCl_2(dppf)CH_2Cl_2$, K_3PO_4, dioxane, 100 °C, 16 h, 34%. (b) $PdCl_2(dppf)CH_2Cl_2$, K_3PO_4, dioxane, 85 °C, 16 h, 65%. (c) Au (111) held at 310 °C and 300 °C for **2** and **3**, respectively.

As shown in Figure S7, a Suzuki-coupling reaction of **4** with commercially available 1,3-bis(4,4,5,5-tetramethyl-1,3,2-dioxaborolan-2-yl)benzene (**5**) or 1,3,5-tris(4,4,5,5-tetramethyl-1,3,2-dioxaborolan-2-yl)benzene (**6**) gave precursors **2p** and **3p** in 34% yield and 65% yield, respectively. Subsequently, these two precursors were applied for the on-surface synthesis of triangulene dimer (**2**) and trimer (**3**).

Synthesis of 9,9'-(3,3",5,5"-tetramethyl-[1,1':3',1"-terphenyl]-4,4"-diyl)dianthracene (2p):

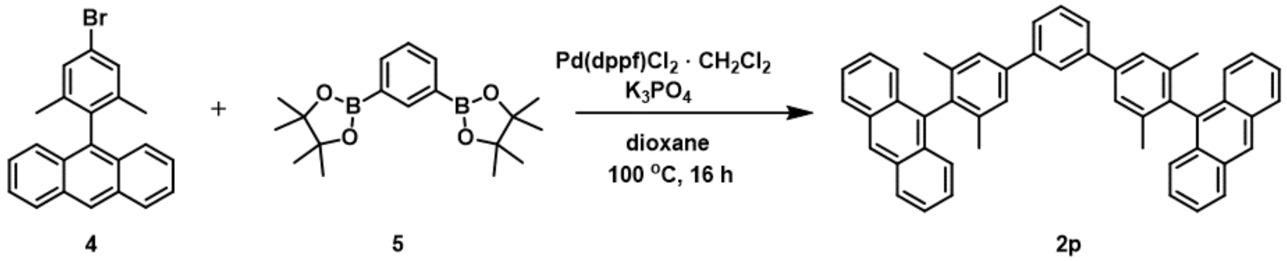

4 5 2p

A 25 mL Schlenk tube charged with 9-(4-bromo-2,6-dimethylphenyl)anthracene (**4**)[6] (50 mg, 0.138 mmol), the commercially available 1,3-bis(4,4,5,5-tetramethyl-1,3,2-dioxaborolan-2-yl)benzene (**5**) (18.3 mg, 0.0554 mmol), [1,1'-bis(diphenylphosphino)ferrocene]dichloropalladium(II) complex with dichloromethane (Pd(dppf)Cl_2•CH_2Cl_2) (11.3 mg, 0.0138 mmol) and K_3PO_4 (176 mg, 0.830 mmol) was evacuated and charged with argon three times. Then degassed 1,4-dioxane (10 mL) was added and the reaction mixture was stirred at 100 °C for 36 h under an argon atmosphere. After cooling to room temperature, the mixture was poured into water and extracted with dichloromethane (DCM) three times. The organic layer was washed with brine and dried over anhydrous $MgSO_4$. The solvent was removed under vacuum. The residue was purified by silica gel column chromatography (iso-hexane: DCM = 5:1) to give compound **2p** as a white solid (30 mg, 34%). ^{1}H NMR (300 MHz, CD_2Cl_2) δ (ppm) = 8.56 (s, 2H), 8.20 - 8.07 (m, 5H), 7.85 - 7.76 (m, 2H), 7.68 (s, 5H), 7.59 - 7.47 (m, 8H), 7.39 (ddd, J = 8.7, 6.5, 1.3 Hz, 4H), 1.85 (s, 12H). ^{13}C NMR (75 MHz, CD_2Cl_2) δ 142.22, 140.99, 138.85, 137.42, 135.82, 132.26, 130.18, 129.91, 129.24, 126.89, 126.87, 126.52, 126.46, 126.40, 126.31, 125.86, 20.50. HR-MALDI-TOF (matrix: DCTB): calc. for [M]$^+$: 638.2969, found for [M]$^+$: 638.2963 (deviation: -0.94 ppm).

Synthesis of 9,9'-(5'-(4-(anthracen-9-yl)-3,5-dimethylphenyl)-3,3",5,5"-tetramethyl -[1,1':3',1"-terphenyl]-4,4"-diyl)dianthracene (3p):

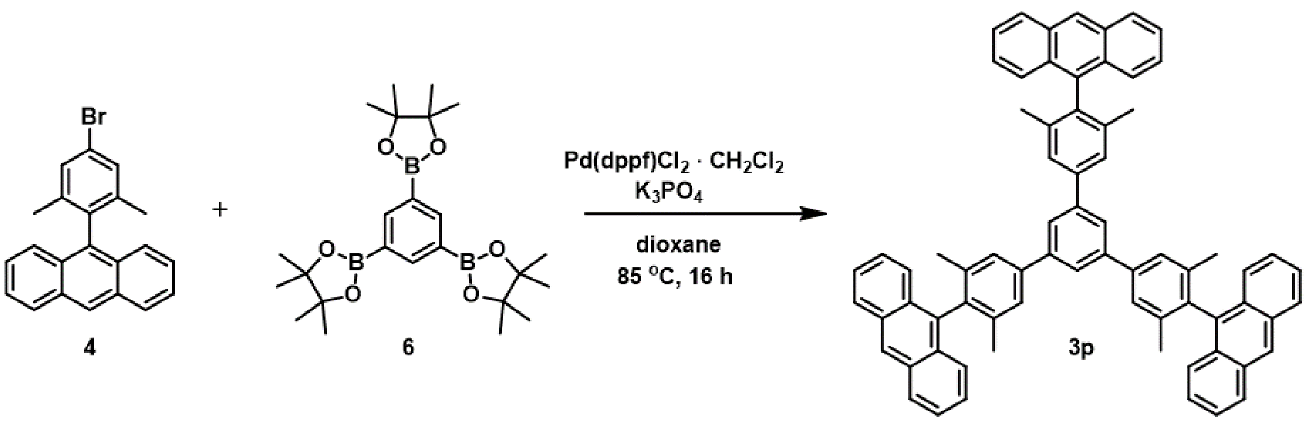

4 6 3p

A 25 mL Schlenk tube charged with compound **4**[6] (26.2 mg, 0.072 mmol), the commercially available **6** (10 mg, 0.022 mmol), [1,1'-bis(diphenylphosphino)ferrocene]dichloropalladium(II) complex with dichloromethane (Pd(dppf)Cl_2•CH_2Cl_2) (3.6 mg, 20% mmol) and K_3PO_4 (42 mg, 0.20 mmol) was evacuated and charged with argon three times. Then degassed 1,4-dioxane (8 mL) was added and the reaction mixture was stirred at 85 ° C for 16 h under an argon atmosphere. After cooling to room temperature, the mixture was poured into water and extracted with dichloromethane (DCM) three times. The organic layer was washed with brine and dried over anhydrous $MgSO_4$. The solvent was removed under vacuum. The residue was purified by silica gel column chromatography (iso-hexane: DCM = 3:1) to give **3p** as a white solid (13 mg, 65%). ^{1}H NMR (300 MHz, $C_2D_2Cl_4$-d_2) δ 8.49 (s, 3H), 8.12 (s, 3H), 8.06 (d, J = 8.4 Hz, 6H), 7.71 (s, 6H), 7.57 – 7.52 (m, 6H), 7.46 (ddd, J = 8.3, 6.6, 1.3 Hz, 6H), 7.35 (ddd, J = 8.7, 6.5, 1.3 Hz, 6H), 1.85 (s, 18H). ^{13}C NMR (76 MHz, $C_2D_2Cl_4$-d_2) δ 142.27, 140.38, 138.34, 136.91, 135.35, 131.57, 129.60, 128.73, 126.55, 125.98, 125.89, 125.40, 125.07, 120.35, 20.47. HR-MALDI-TOF (matrix: DCTB): calc. for [M]$^+$: 918.4220, found for [M]$^+$: 918.4221 (deviation: -0.1 ppm).

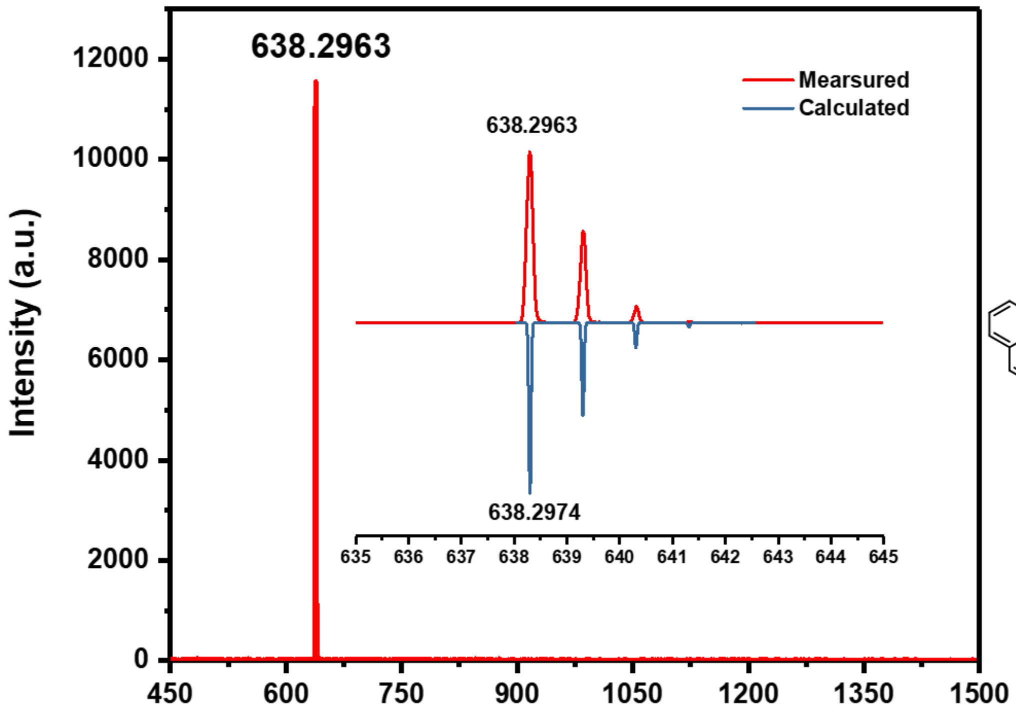

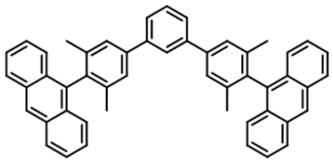

2p

Figure S9. Liquid-state HR-MALDI-TOF-MS of **2p** (matrix: DCTB)

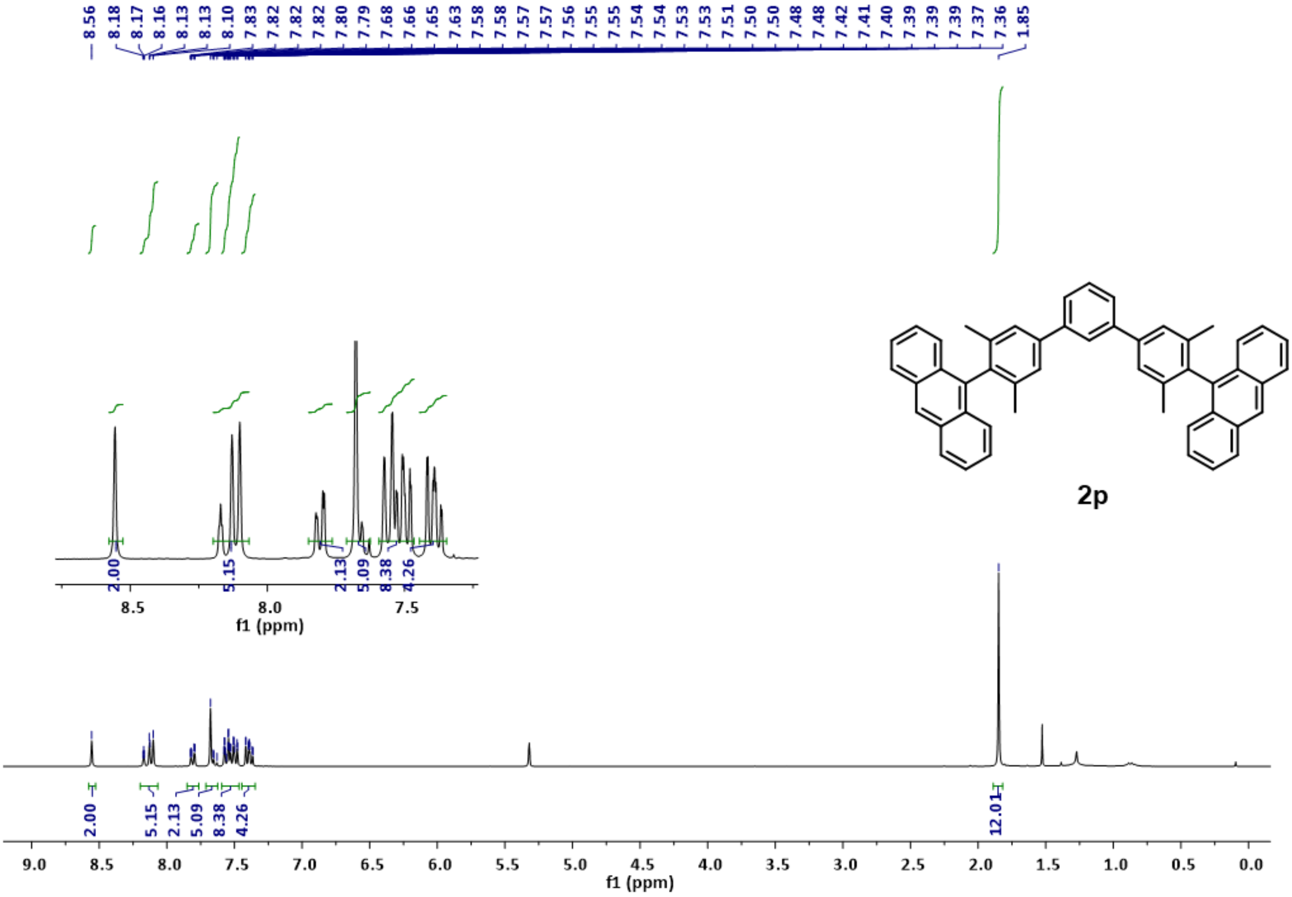

Figure S10. ^{1}H-NMR spectrum of **2p** dissolved in CD_2Cl_2, 300 MHz, 296 K.

References

[1] E. Turco, A. Bernhardt, N. Krane, L. Valenta, R. Fasel, M. Juríček, P. Ruffieux, *JACS Au* **2023**, *3*, 1358.

[2] M. Ternes, *New J. Phys.* **2015**, *17*, 063016.

[3] R. Ortiz, G. Catarina, J. Fernández-Rossier, *2D Mater.* **2023**, *10*, 015015.

[4] B. Sutherland, *Phys. Rev. B* **1986**, *34*, 5208.

[5] D. Jacob, J. Fernández-Rossier, *Phys. Rev. B* **2022**, *106*.

[6] G. Catarina, J. C. G. Henriques, A. Molina-Sánchez, A. T. Costa, J. Fernández-Rossier, *Phys. Rev. Res.* **2023**, *5*, 043226.

[7] S. Mishra, D. Beyer, K. Eimre, R. Ortiz, J. Fernández-Rossier, R. Berger, O. Gröning, C. A. Pignedoli, R. Fasel, X. Feng, P. Ruffieux, *Angew. Chem. Int. Ed.* **2020**, *59*, 12041.

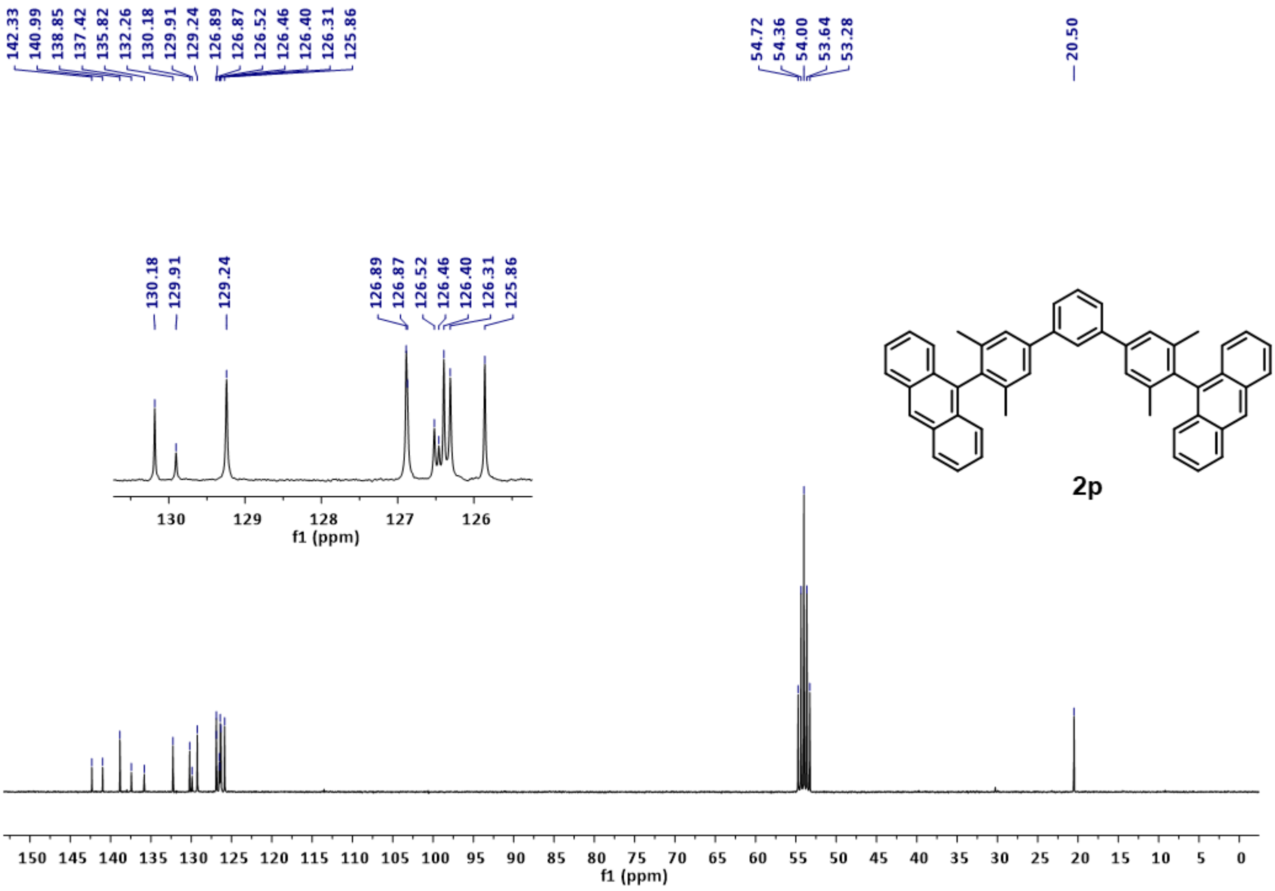

Figure S11. ^{13}C-NMR spectrum of **2p** dissolved in CD_2Cl_2, 75 MHz, 296 K.

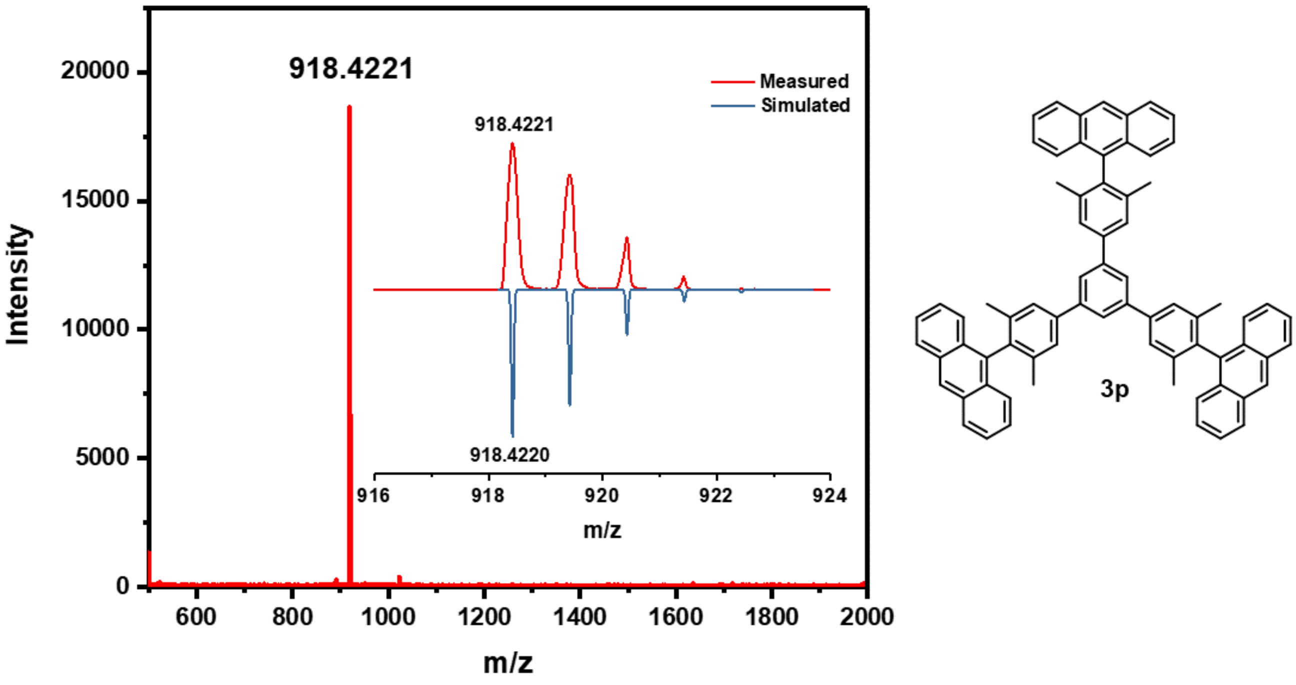

Figure S12. Liquid-state HR-MALDI-TOF-MS of **3p** (matrix: DCTB)

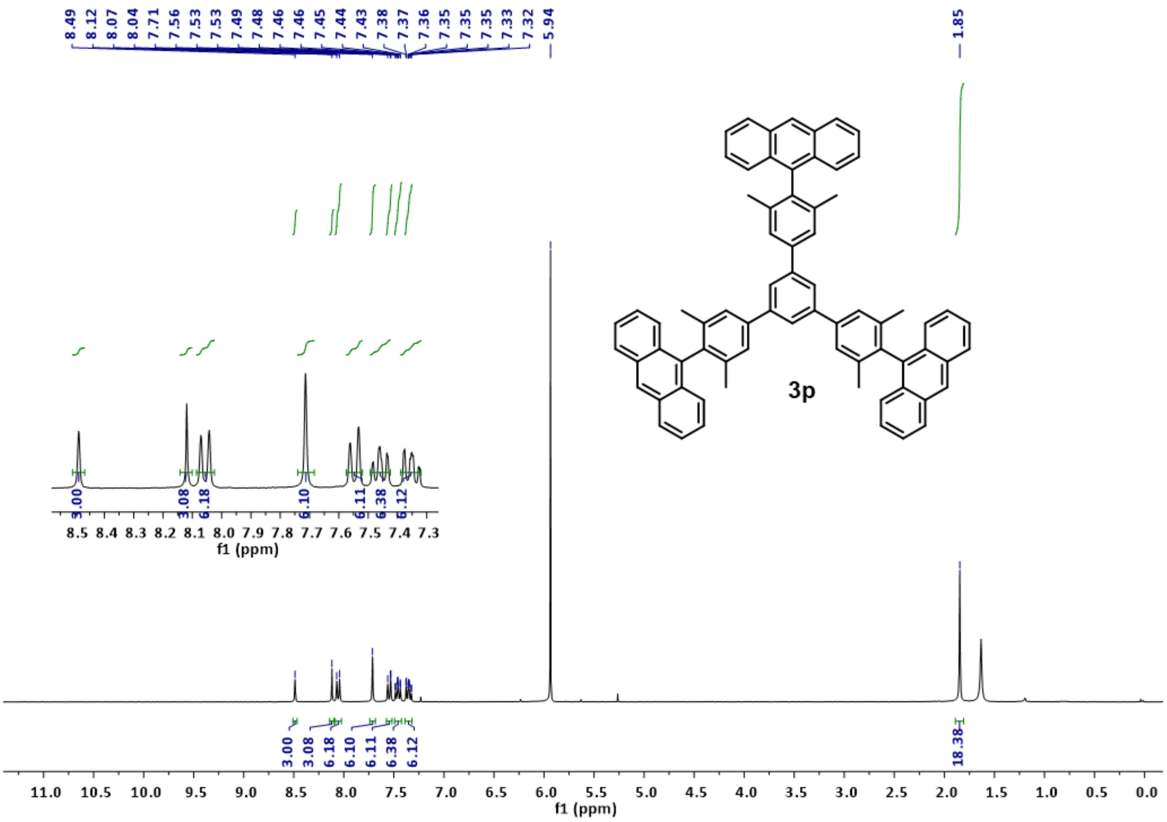

Figure S13. ^{1}H-NMR spectrum of **3p** dissolved in $C_2D_2Cl_4$-d_2, 300 MHz, 296 K

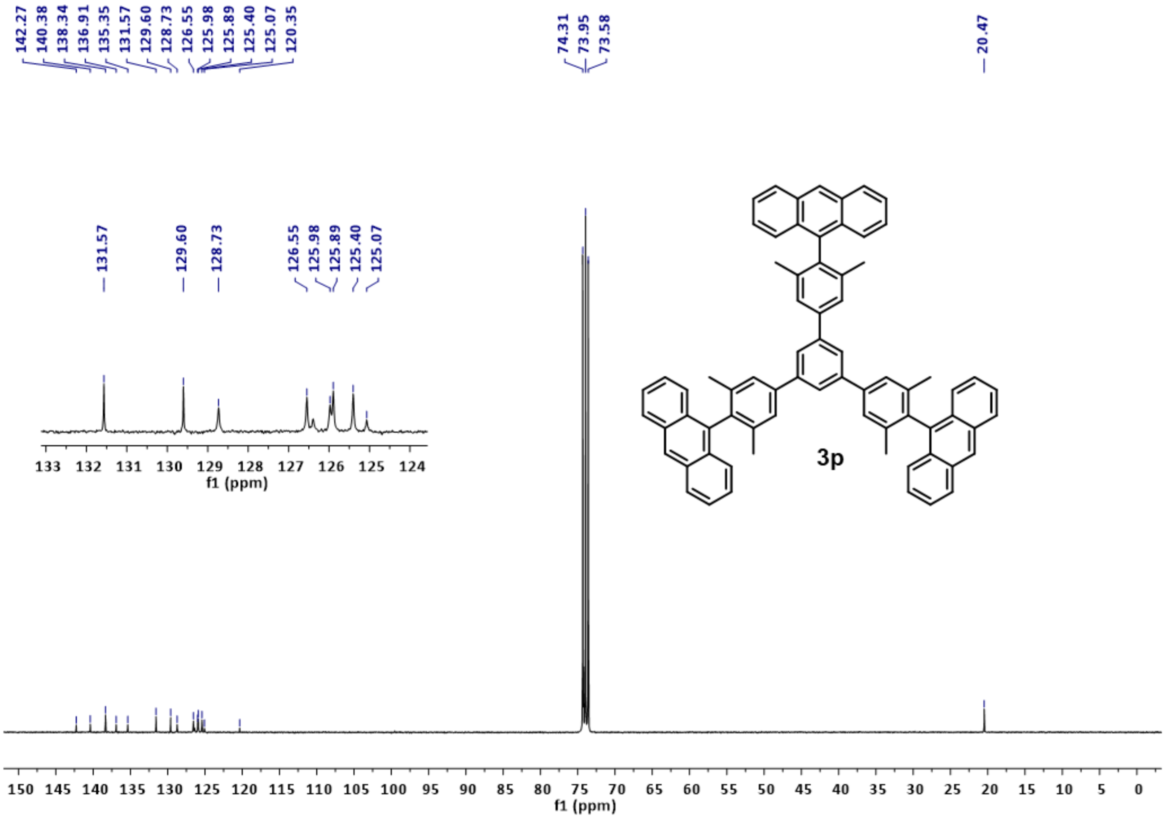

Figure S14. ^{13}C-NMR spectrum of **3p** dissolved in $C_2D_2Cl_4$-d_2, 75 MHz, 296 K.
